# Multi-Omics Analysis and In Vitro Experimental Validation Identify Candidate Mechanisms of Baicalein Against Chronic Obstructive Pulmonary Disease

**DOI:** 10.3390/molecules31101610

**Published:** 2026-05-11

**Authors:** Yinan Liu, Xuhua Yuan, Wei Shi, Zhidong Qiu, Xuelian Dong

**Affiliations:** School of Pharmacy, Changchun University of Chinese Medicine, No. 1035 Boshuo Road, Jingyue National High-Tech Industrial Development Zone, Changchun 130117, China; 24204895142@stu.ccucm.edu.cn (Y.L.); 16643036877@163.com (X.Y.); 23203089506@stu.ccucm.edu.cn (W.S.); qzdcczy@163.com (Z.Q.)

**Keywords:** baicalein, COPD, chronic obstructive pulmonary disease, macrophage-associated immune regulation, CD163, inflammation, target prioritization, multi-omics analysis, BEAS-2B

## Abstract

Chronic obstructive pulmonary disease (COPD) is characterized by persistent airflow limitation, chronic airway inflammation, and immune dysregulation, and currently available therapies remain insufficient to effectively halt disease progression. In this study, we used an integrative, hypothesis-generating strategy to investigate the potential mechanisms of baicalein against COPD by combining multi-dataset transcriptomic analysis, single-cell transcriptomics, machine learning-based feature selection, Mendelian randomization (MR), molecular simulation, virtual knockout analysis, and in vitro validation. Putative targets of baicalein were predicted using CTD, SEA, and SwissTargetPrediction, and were intersected with COPD-related genes collected from GeneCards and OMIM. Four GEO datasets (GSE20257, GSE42057, GSE76925, and GSE130928) were integrated after batch-effect correction, yielding a combined cohort of 260 control samples and 250 COPD samples. Candidate genes were prioritized by intersecting the results of LASSO regression, random forest, and support vector machine. Immune-cell infiltration was estimated using CIBERSORT, and single-cell transcriptomic data were used to define the cellular localization of prioritized genes. Formal protein-level MR analysis was conducted for CD163 using deCODE plasma protein pQTL/GWAS summary statistics as the exposure dataset and the IEU OpenGWAS COPD dataset (ebi-a-GCST90018807) as the outcome dataset. Molecular docking, molecular dynamics simulation, and virtual knockout analysis were further used to provide structural and network-level supportive evidence. Finally, LPS-stimulated BEAS-2B cells were used as an epithelial inflammatory model to evaluate the effects of baicalein by CCK-8 assay, wound-healing assay, ELISA, and RT-qPCR. Five core genes were prioritized, namely ABCC1, CD163, CYP1B1, IKBKB, and PIK3CA. Immune infiltration and single-cell analyses suggested that macrophage-associated immune regulation may represent an important mechanistic direction. MR analysis provided supportive genetic evidence for prioritizing CD163 in COPD. Molecular simulation offered preliminary structural support for several target-compound interactions. In LPS-stimulated BEAS-2B cells, baicalein reduced inflammatory cytokine release and modulated the expression of IKBKB, PIK3CA, IL1B, IL6, and IL10, thereby providing epithelial-level support for the predicted network. Taken together, these findings suggest that baicalein may exert anti-inflammatory effects in COPD through a multi-target, immune-associated mechanism, with macrophage-related regulation and CD163 emerging as noteworthy candidate directions for further investigation. This study provides an integrative framework for target prioritization and mechanistic exploration, while the predicted macrophage-centered mechanisms still require dedicated validation in immune-cell and in vivo models.

## 1. Introduction

Chronic obstructive pulmonary disease (COPD) is a common and progressive respiratory disorder characterized by persistent airflow limitation, chronic airway inflammation, and structural lung injury, and is clinically associated with mucus hypersecretion, impaired pulmonary function, and epithelial barrier disruption [1,2]. Owing to its high prevalence, disability burden, and mortality, COPD has become one of the leading causes of death worldwide and remains a major global public health challenge [3,4]. Current clinical management of COPD mainly relies on symptomatic interventions, including bronchodilators, corticosteroids, and anti-infective therapy. Although these approaches can alleviate symptoms and partially delay disease progression, they do not effectively halt the underlying pathological process and may also be accompanied by adverse effects with long-term use [5,6,7]. Therefore, identifying biologically relevant molecular targets involved in chronic inflammation and immune dysregulation, and exploring candidate bioactive compounds with multi-target regulatory potential, may provide new opportunities for COPD intervention.

COPD is a biologically heterogeneous disease in which genetic susceptibility, environmental exposure, chronic inflammation, and aberrant tissue repair interact across multiple cell types and signaling pathways. In this context, innate immune cells, particularly macrophages, play central roles in inflammatory amplification, cytokine release, and the imbalance between tissue injury and repair [8,9]. At the same time, airway epithelial cells are not merely passive structural barriers, but active participants in inflammatory signaling, immune-cell communication, and pathological remodeling. Thus, COPD should be understood as a multicellular disease involving extensive crosstalk between immune cells and structural cells. In addition, its pathogenesis is not restricted to a single inflammatory pathway. Rather, multiple immune-regulatory and inflammation-related pathways, including but not limited to PI3K-Akt, NF-κB, TNF, and JAK-STAT signaling, are involved in shaping the disease network [10]. Therefore, examining immune-cell infiltration patterns, cellular localization of key genes, and candidate regulatory nodes may help to more precisely define biologically meaningful intervention directions in COPD.

Baicalein is a natural flavonoid mainly derived from medicinal plants such as *Scutellaria baicalensis* and has attracted increasing attention because of its anti-inflammatory, antioxidant, immunomodulatory, and respiratory protective activities [11,12]. Previous studies in pulmonary inflammatory settings have shown that baicalein can alleviate inflammatory injury and may be associated with pathways such as PI3K-Akt, TNF, and NF-κB [13]. In addition, baicalein has been reported to influence macrophage-related immune processes, including macrophage phenotype regulation and inflammation-related factor expression, thereby suggesting a possible role in immune-microenvironment modulation [14]. However, like many natural products, baicalein is likely to act through multi-target and multi-pathway mechanisms, and reliance on a single database, a single analytical layer, or a single cohort may be insufficient for robust target prioritization. For this reason, an integrative framework is needed in which different analytical layers provide complementary evidence: multi-cohort transcriptomics improves the robustness of disease-associated expression signals, machine learning refines candidate features, immune infiltration and single-cell analysis provide immune and cellular context, Mendelian randomization offers supportive genetic evidence, molecular docking and molecular dynamics simulation provide preliminary structural support, and virtual knockout analysis adds systems-level information on downstream network perturbation [15,16,17]. Importantly, these computational approaches are inherently predictive rather than conclusive and are best interpreted as tools for target prioritization and mechanistic hypothesis generation.

Based on this rationale, the present study adopted an integrative strategy to investigate the potential mechanisms of baicalein against COPD. Multi-cohort transcriptomic datasets from GEO were integrated after batch-effect correction, and LASSO regression, random forest, and support vector machine were jointly applied to prioritize robust candidate genes. CIBERSORT and single-cell transcriptomic analysis were further used to define immune-cell infiltration patterns and the cellular localization of prioritized genes. In addition, Mendelian randomization, molecular docking, molecular dynamics simulation, and virtual knockout analysis were performed to provide complementary genetic, structural, and network-level support for target prioritization. Finally, in vitro experiments were conducted in an LPS-stimulated BEAS-2B epithelial inflammatory model to evaluate cell viability, migration-related phenotype, inflammatory cytokine secretion, and expression changes in selected target-related genes. Through this multi-layer approach, we aimed to identify biologically plausible targets and candidate mechanistic directions associated with baicalein in COPD, while providing a basis for subsequent validation in immune-cell and in vivo models.

## 2. Results

### 2.1. Network Pharmacology and Machine Learning Analysis of Baicalein in COPD

#### 2.1.1. Prediction of Drug- and Disease-Related Targets

**Figure 1:**
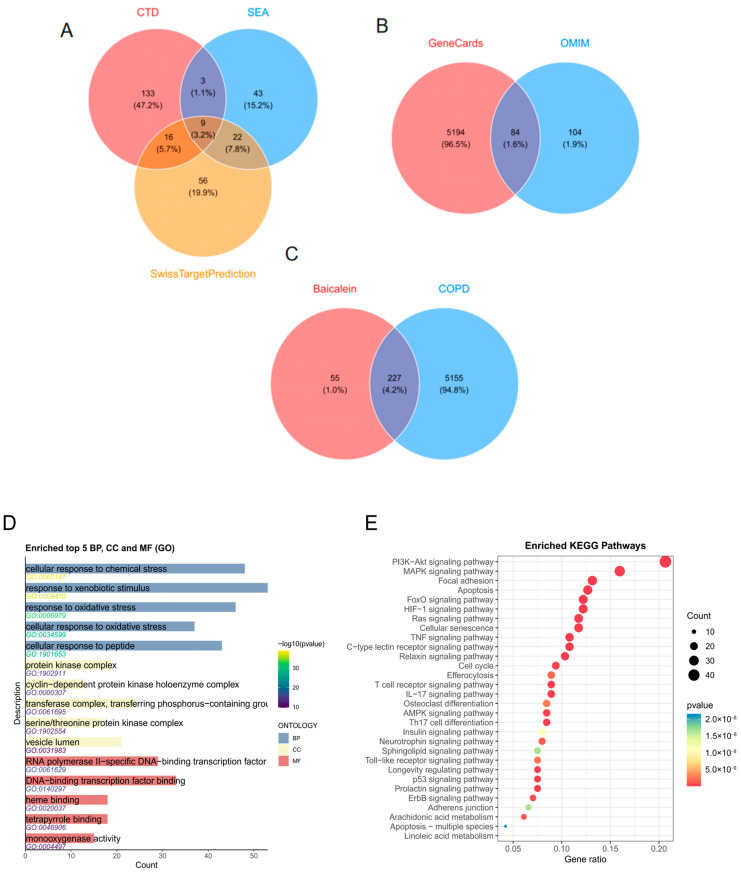
Drug–disease target prediction and functional enrichment analysis of baicalein against COPD. (**A**) Venn diagram showing the candidate targets of baicalein predicted from the CTD, SEA, and SwissTargetPrediction databases, with 282 unique targets obtained after merging and removing duplicates. Percentages were calculated based on the total number of unique targets and may not sum exactly to 100% due to rounding. (**B**) COPD-related targets were collected from the GeneCards and OMIM databases, resulting in 5382 unique disease-associated targets. (**C**) A total of 227 overlapping targets between baicalein and COPD were identified and used for subsequent enrichment analysis. (**D**) Gene Ontology (GO) enrichment analysis of the overlapping targets, including biological process (BP), cellular component (CC), and molecular function (MF) terms. The enriched GO terms were mainly associated with responses to chemical or xenobiotic stimuli, protein kinase complexes, and transcription factor-related molecular functions. (**E**) Kyoto Encyclopedia of Genes and Genomes (KEGG) pathway enrichment analysis of the overlapping targets. The bubble plot indicates that these targets were mainly enriched in pathways such as PI3K-Akt signaling, MAPK signaling, focal adhesion, and apoptosis. In the KEGG bubble plot, dot size represents the number of enriched genes, and color indicates the enrichment significance.

Potential targets of baicalein were predicted using the CTD, SEA, and SwissTargetPrediction databases, yielding 161, 77, and 103 candidate targets, respectively. After taking the union of these datasets, a total of 282 candidate targets of baicalein were obtained (Figure 1A). For COPD-related targets, 5278 targets were predicted from GeneCards and 188 from OMIM. After merging and removing duplicates, a total of 5382 COPD-related targets were identified (Figure 1B). The intersection between the candidate target set of baicalein and the COPD-related target set yielded 227 overlapping targets (Figure 1C), suggesting that baicalein may participate in the regulation of COPD-related molecular networks through multiple targets.

#### 2.1.2. Enrichment Analysis

The GO enrichment analysis is shown in Figure 1D. These genes were mainly enriched in biological processes such as cellular response to chemical stress and response to xenobiotic stimulus; in cellular components such as the protein kinase complex and cyclin-dependent protein kinase holoenzyme complex; and in molecular functions such as RNA polymerase II-specific DNA-binding transcription factor activity and DNA-binding transcription factor binding. KEGG pathway analysis (Figure 1E) further showed that these genes were significantly enriched in pathways including the PI3K-Akt signaling pathway, MAPK signaling pathway, focal adhesion, and apoptosis.

#### 2.1.3. Machine Learning-Based Screening of Feature Genes

**Figure 2:**
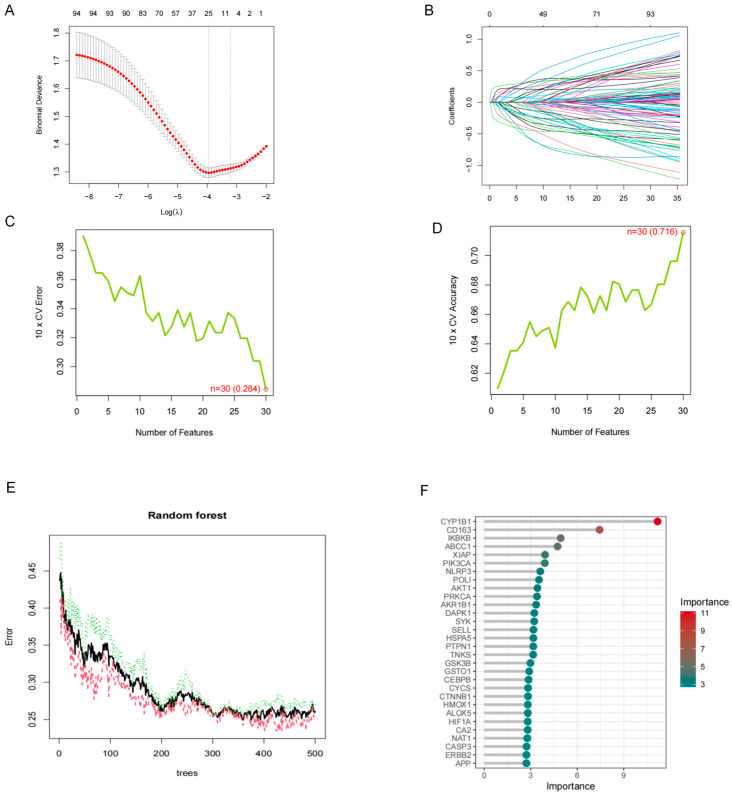
Machine learning-based screening of feature genes from the 227 overlapping targets. (**A**) Cross-validation curve of the LASSO model showing the relationship between binomial deviance and log(λ), used to determine the optimal penalty parameter. Red dots represent the mean cross-validated binomial deviance, grey error bars represent standard errors, and vertical dashed lines indicate the optimal λ values.(**B**) Coefficient profile plot of the LASSO model. Each colored line represents the coefficient trajectory of one gene as the regularization parameter changes. Based on the LASSO analysis, 25 feature genes were identified. (**C**) SVM-RFE analysis showing the relationship between the number of selected features and the 10-fold cross-validation error. The green line represents the cross-validation error, and the red circle indicates the optimal number of selected features.(**D**) SVM-RFE analysis showing the relationship between the number of selected features and the 10-fold cross-validation accuracy. The green line represents the cross-validation accuracy, and the red circle indicates the optimal number of selected features. According to the SVM-RFE results, 30 feature genes were selected. (**E**) Error-rate plot of the random forest (RF) model as the number of trees increased, showing the variation in model error during the training process. The black line represents the overall out-of-bag error rate, whereas the colored dashed lines represent class-specific error rates. (**F**) Variable importance plot of the RF model. Genes with higher importance scores contributed more strongly to feature selection, and 17 feature genes were ultimately identified by the RF model. The color gradient indicates the relative importance score of each gene, and grey horizontal lines are used to guide gene ranking.

**Figure 3:**
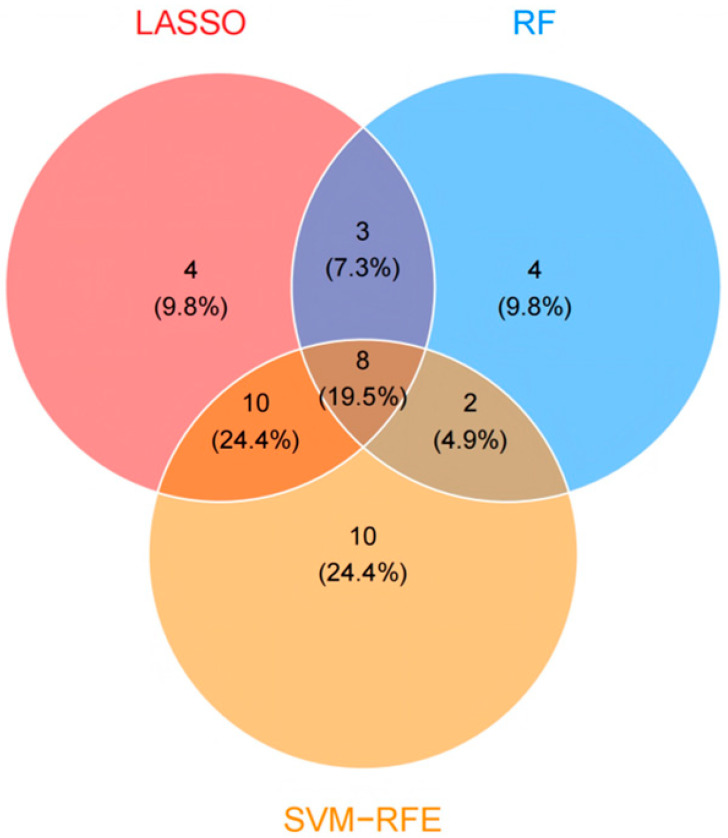
Intersection of feature genes identified by three machine learning algorithms. A Venn diagram was used to visualize the overlap among the feature genes identified by LASSO, random forest (RF), and SVM-recursive feature elimination (SVM-RFE). By intersecting the results of the three algorithms, eight common feature genes were obtained. These shared genes were considered stable candidate genes for subsequent analyses. Percentages were calculated based on the total number of unique targets and may not sum exactly to 100% due to rounding.

To further identify relevant feature genes, LASSO, random forest (RF), and SVM-RFE were applied to the expression matrix of the 227 overlapping genes. LASSO identified 25 feature genes (Figure 2A,B), SVM-RFE identified 30 feature genes (Figure 2C,D), and RF identified 17 feature genes (Figure 2E,F). By intersecting the results of the three algorithms, eight stable feature genes were ultimately obtained (Figure 3).

#### 2.1.4. Analysis of Differential Expression, Chromosomal Localization, and Expression Correlation of the Core Genes

**Figure 4:**
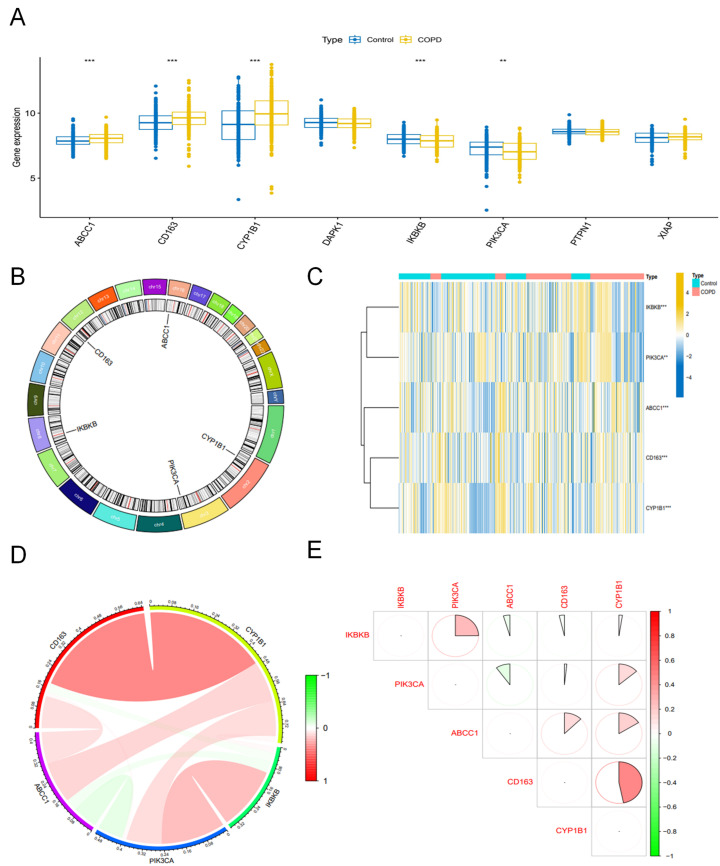
Differential expression, chromosomal localization, and correlation analysis of the core genes. Eight stable feature genes (ABCC1, CD163, CYP1B1, DAPK1, IKBKB, PIK3CA, PTPN1, and XIAP) were obtained from the intersection of the three machine learning algorithms. (**A**) Box plots showing the expression levels of these eight genes in the control and COPD groups. Based on differential expression analysis, five genes (ABCC1, CD163, CYP1B1, IKBKB, and PIK3CA) met the screening criteria and were retained as core genes, whereas DAPK1, PTPN1, and XIAP were excluded. Among the retained genes, ABCC1, CD163, and CYP1B1 were highly expressed in the COPD group, while IKBKB and PIK3CA were highly expressed in the control group. (**B**) Circos plot showing the chromosomal locations of the five core genes. (**C**) Heatmap of the expression patterns of the five core genes in the integrated transcriptomic dataset. (**D**,**E**) Correlation analyses among the five core genes, showing mainly positive correlations in the COPD group. Statistical significance is indicated as ** *p* < 0.01, *** *p* < 0.001.

By intersecting the results of the three machine learning algorithms, eight stable feature genes were identified, namely ABCC1, CD163, CYP1B1, DAPK1, IKBKB, PIK3CA, PTPN1, and XIAP. To further prioritize the core genes, differential expression analysis was performed on these eight overlapping genes based on the integrated transcriptomic data. The results showed that five genes—ABCC1, CD163, CYP1B1, IKBKB, and PIK3CA—met the predefined differential expression criteria and were therefore retained as the final core genes, whereas DAPK1, PTPN1, and XIAP were excluded because they did not show significant differential expression. Among the five retained genes, IKBKB and PIK3CA were highly expressed in the control group, whereas ABCC1, CD163, and CYP1B1 were highly expressed in the COPD group. These results are shown in the box plots and heatmaps (Figure 4A,C). The chromosomal locations of the five core genes are presented in Figure 4B. Correlation analysis further demonstrated strong correlations among the five core genes in the COPD group, predominantly positive correlations (Figure 4D,E).

#### 2.1.5. SHAP Analysis and Nomogram Construction

**Figure 5:**
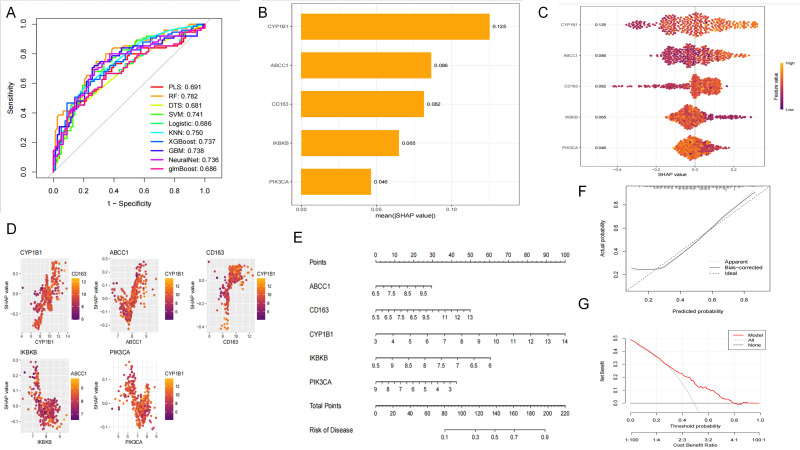
SHAP analysis and nomogram construction based on the core genes. The expression levels of the five core genes (ABCC1, CD163, CYP1B1, IKBKB, and PIK3CA) were used to construct 10 machine learning models, including PLS, RF, DT, SVM, Logistic, KNN, XGBoost, GBM, NeuralNet, and glmBoost. (**A**) Receiver operating characteristic (ROC) curves of the 10 machine learning models. Among these models, the random forest (RF) model showed the highest area under the curve (AUC = 0.782), indicating the best discriminatory performance based on ROC analysis. (**B**) SHAP feature importance plot for the RF model, ranking the contribution of the five core genes. (**C**) SHAP summary plot showing the overall impact of each gene on the model output. (**D**) SHAP dependence plots illustrating the effects of individual core genes on the RF model prediction. Based on the selected core genes, a nomogram was established to predict COPD risk (**E**), and its predictive performance and potential clinical utility were further evaluated by calibration (**F**) and decision curve analysis (**G**).

To further evaluate the predictive performance of the five core genes, their expression levels were extracted and used to construct 10 machine learning models, including PLS, RF, DT, SVM, Logistic, KNN, XGBoost, GBM, NeuralNet, and glmBoost. The results showed that all 10 machine learning models exhibited good diagnostic value based on the core genes, among which the RF model demonstrated the highest diagnostic stability (Figure 5A). SHAP analysis indicated that the contributions of the core genes to the RF model, in descending order, were CYP1B1, ABCC1, CD163, IKBKB, and PIK3CA (Figure 5B–D). Based on these findings, a nomogram was constructed, and its consistency and potential clinical benefit were evaluated using calibration and decision curves (Figure 5E–G).

#### 2.1.6. Immune Infiltration Analysis

**Figure 6:**
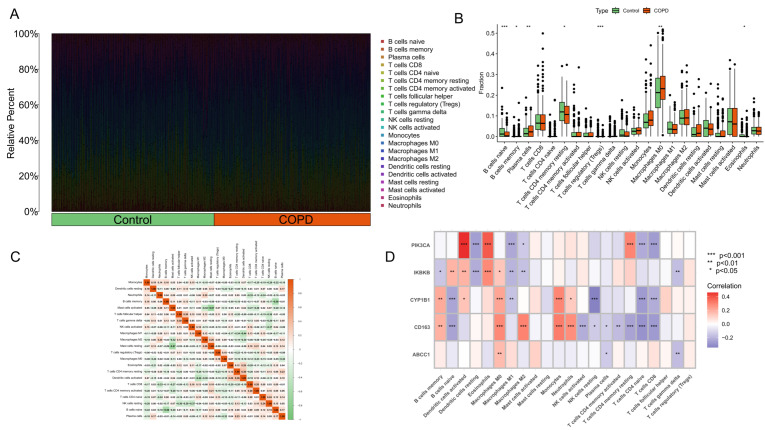
Immune infiltration analysis in control and COPD samples. The CIBERSORT algorithm was used to estimate the relative proportions of infiltrating immune cells in the integrated transcriptomic dataset: (**A**) Stacked bar plot showing the relative proportions of 22 immune cell subsets estimated by CIBERSORT in individual samples from the control and COPD groups. Each vertical bar represents one sample, and different colors indicate distinct immune cell subsets. (**B**) Comparison of immune cell infiltration between the two groups. Naive B cells, resting memory CD4 T cells, and eosinophils were more abundant in the control group, whereas plasma cells and M0 macrophages were more abundant in the COPD group. (**C**) Correlation matrix showing the relationships among different immune cell subsets. (**D**) Heatmap showing the correlations between the five core genes and immune cell infiltration. PIK3CA was positively correlated with activated dendritic cells, eosinophils, and activated memory CD4 T cells, whereas CD163 showed positive correlations with M0 macrophages, M2 macrophages, monocytes, neutrophils, and memory B cells. Statistical significance is indicated as * *p* < 0.05, ** *p* < 0.01, *** *p* < 0.001.

To explore the mechanisms underlying the differences between the COPD and control groups at multiple levels, the CIBERSORT algorithm was used to analyze and visualize the types and proportions of immune cells in each sample (Figure 6A). Subsequently, by comparing differences in immune infiltration scores between the two groups, immune cell populations with statistically significant differences between the control and COPD groups were identified (Figure 6B). The results showed that naive B cells, resting memory CD4 T cells, and eosinophils were significantly more active in the control group, whereas plasma cells and M0 macrophages were significantly more active in the COPD group. Heatmaps were further used to visualize the internal correlations among ic perturbation experiments.

With respect to experimental immune cell subsets and to analyze the correlations between core genes and immune cells (Figure 6C,D), the results showed that PIK3CA was positively correlated with activated dendritic cells, eosinophils, and activated memory CD4 T cells, but negatively correlated with CD8 T cells and other immune cell subsets. IKBKB was positively correlated with eosinophils and negatively correlated with activated dendritic cells and related cell populations. CYP1B1 was positively correlated with monocytes and negatively correlated with resting NK cells, naive B cells, and naive CD4 T cells. CD163 was positively correlated with M0 macrophages, M2 macrophages, monocytes, neutrophils, and memory B cells, but negatively correlated with naive B cells, naive CD4 T cells, and CD8 T cells.

#### 2.1.7. Single-Cell Analysis

**Figure 7:**
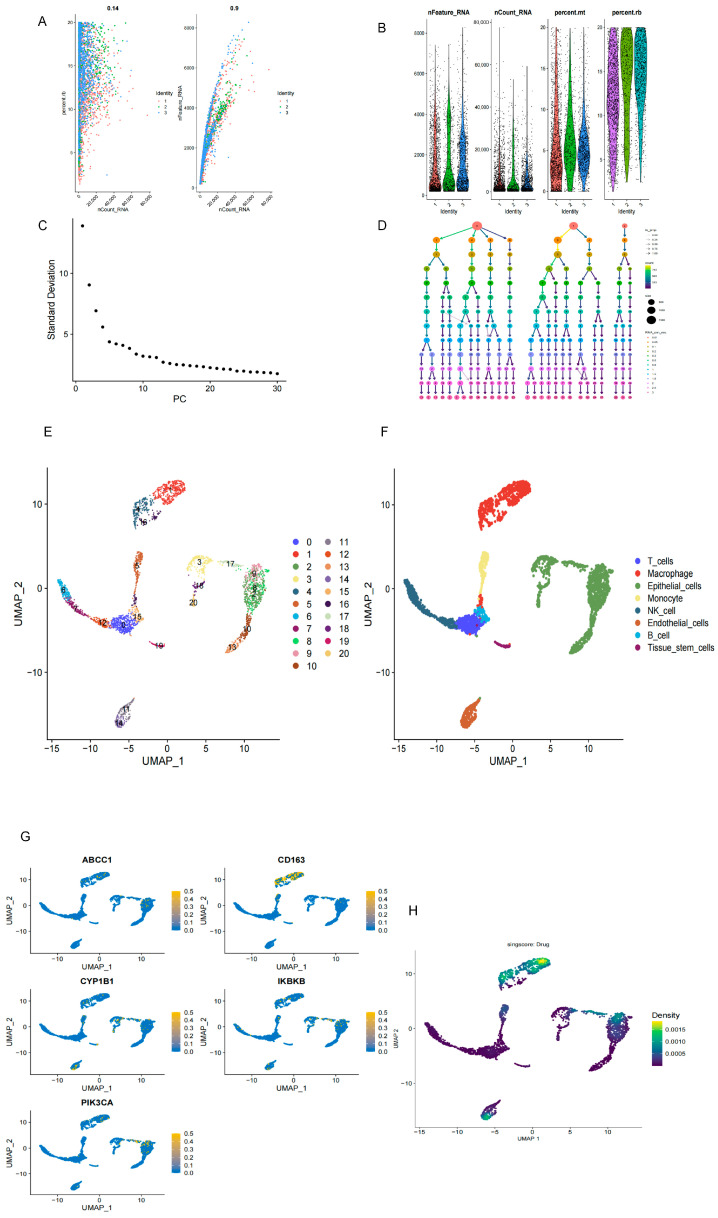
Single-cell transcriptomic analysis of the core genes in chronic obstructive pulmonary disease (COPD). Single-cell RNA-sequencing dataset GSE167295 was subjected to quality control, clustering, and cell-type annotation: (**A**) Scatter plots showing the relationships between nCount_RNA and percent.mt, and between nCount_RNA and nFeature_RNA in single cells. (**B**) Violin plots presenting the distributions of quality-control metrics across cell clusters, including nFeature_RNA, nCount_RNA, percent.mt, and percent.rb. (**C**) Elbow plot used to determine the number of principal components for downstream analysis. (**D**) Cluster tree showing clustering stability under different resolution parameters; PC = 10 and a resolution of 1.2 were selected for subsequent analysis. (**E**) UMAP plot showing 21 unsupervised cell clusters identified in the single-cell RNA-seq dataset. (**F**) UMAP plot showing the annotation of these clusters into eight major cell populations, including T cells, macrophages, epithelial cells, monocytes, NK cells, endothelial cells, B cells, and tissue stem cells. (**G**) Feature plots showing the single-cell expression patterns of the five core genes (ABCC1, CD163, CYP1B1, IKBKB, and PIK3CA). (**H**) UCell scoring plot showing that the core gene set was predominantly active in macrophages, suggesting that macrophage-associated immune regulation may represent an important cellular context for the core genes in COPD.

After quality control, the single-cell dataset GSE167295 was subjected to clustering and annotation analysis. As shown in Figure 7A,B, a positive correlation was observed between the number of detected genes and the measured gene expression level per cell. Low-quality cells and cells with high mitochondrial or ribosomal gene expression were excluded to ensure data quality. Based on the ElbowPlot (Figure 7C) and cluster tree (Figure 7D), PC = 10 and a resolution of 1.2 were selected for downstream clustering analysis. UMAP identified 21 cell clusters (Figure 7E), which were subsequently annotated into eight major cell populations, including T cells, macrophages, epithelial cells, monocytes, NK cells, endothelial cells, B cells, and tissue stem cells (Figure 7F). Further single-cell analysis showed that the prioritized core genes were predominantly localized in the macrophage population (Figure 7G). Consistently, UCell scoring indicated that the core gene set was mainly active in macrophages (Figure 7H). These findings suggest that macrophage-associated immune regulation may represent an important mechanistic direction in COPD and provide cellular-context support for the prioritized core genes identified in the integrated analysis.

#### 2.1.8. Plasma Protein Mendelian Randomization Analysis

**Figure 8:**
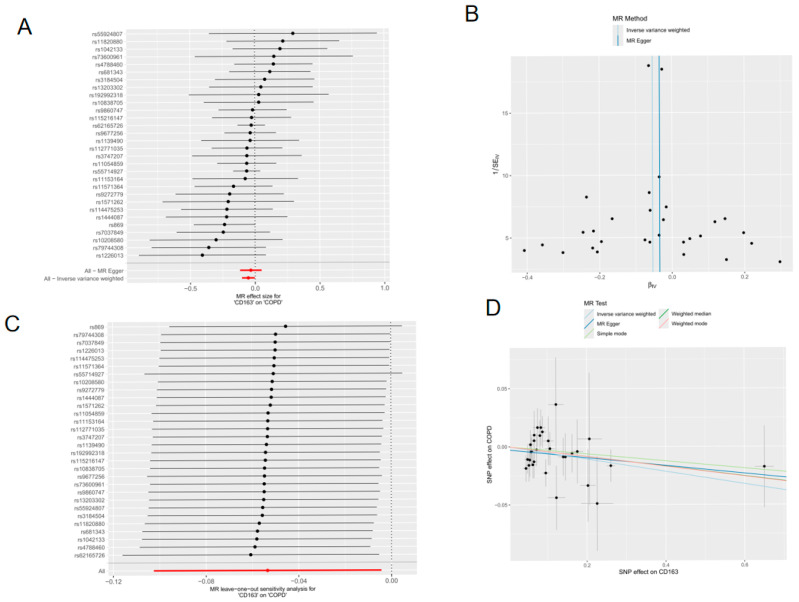
Mendelian randomization analysis of plasma CD163 protein levels and COPD risk. Protein-level Mendelian randomization (MR) analysis was performed to evaluate the association between genetically predicted plasma CD163 levels and the risk of chronic obstructive pulmonary disease (COPD). Thirty single-nucleotide polymorphisms (SNPs) were retained as instrumental variables after harmonization, and the inverse variance weighted (IVW) method was used as the primary MR approach, with MR-Egger, weighted median, simple mode, and weighted mode methods applied as complementary analyses. (**A**) Forest plot showing the MR effect estimates for individual SNPs and the overall estimate. Red lines indicate the pooled MR estimates based on all retained SNPs. (**B**) Funnel plot assessing the symmetry of SNP-specific MR estimates. (**C**) Leave-one-out sensitivity analysis showing that the association was not driven by any single SNP. The red line indicates the overall IVW estimate. (**D**) Scatter plot showing the relationship between SNP effects on CD163 and SNP effects on COPD across different MR methods. The IVW analysis indicated a significant association between genetically predicted plasma CD163 levels and COPD risk (OR = 0.948, 95% CI: 0.903–0.996, *p* = 0.033), providing supportive genetic evidence for prioritizing CD163 as a COPD-related protein target.

In this study, formal protein-level Mendelian randomization (MR) analysis was ultimately conducted only for CD163. Five MR methods were applied to evaluate the association between genetically predicted CD163 plasma protein levels and the risk of chronic obstructive pulmonary disease (COPD), with the inverse variance weighted (IVW) method used as the primary analysis. As shown in Figure 8, the IVW results indicated a significant association between CD163 and COPD risk (beta = −0.053482, SE = 0.025043, *p* = 0.032712; OR = 0.947923, 95% CI: 0.902518–0.995612). After harmonization, 30 SNPs were retained as instrumental variables, and all candidate exposure instruments had F-statistics > 10 (range: 29.86–739.95; mean: 100.32), suggesting limited risk of weak instrument bias. The MR-Egger intercept test did not detect significant directional horizontal pleiotropy (intercept = −0.002731, SE = 0.004793, *p* = 0.573366). In addition, Cochran’s Q test did not indicate significant heterogeneity in either the MR-Egger model (Q = 19.494579, *p* = 0.882184) or the IVW model (Q = 19.819235, *p* = 0.898457). The funnel plot was approximately symmetric, and leave-one-out analysis showed that sequential removal of each SNP yielded consistent results, indicating that the observed association was not driven by a single SNP.

However, formal bidirectional MR/directionality testing was not incorporated in the present study, and reverse-causation exclusion, therefore, remains incomplete. In addition, MR-PRESSO and colocalization analyses were not performed. Accordingly, the MR findings should be interpreted as supportive genetic evidence for prioritizing CD163 rather than definitive proof of causality.

#### 2.1.9. Molecular Docking

**Figure 9:**
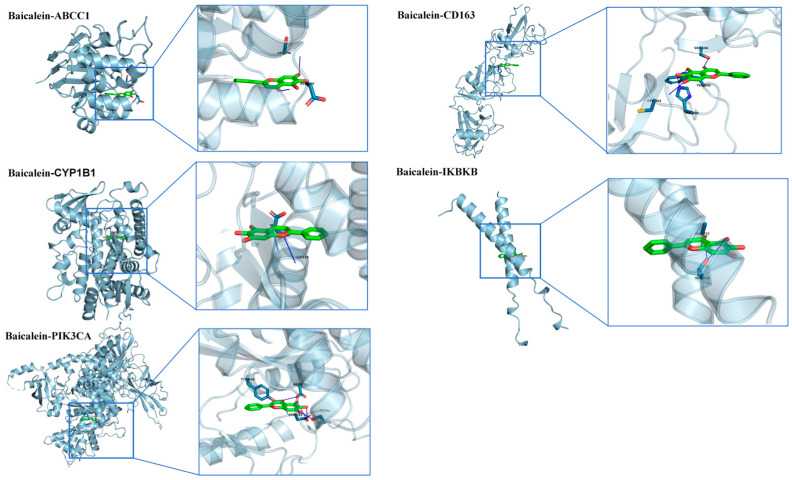
Molecular docking analysis of baicalein with the core target proteins. The docking conformations of baicalein with the five core target proteins are shown, including ABCC1, CD163, CYP1B1, IKBKB, and PIK3CA. For each target, the overall protein–ligand binding mode and an enlarged view of the binding pocket are presented. The docking results suggested that baicalein could bind to all five core proteins, with binding energies of −7.693 kcal/mol for ABCC1, −7.958 kcal/mol for CD163, −10.350 kcal/mol for CYP1B1, −5.717 kcal/mol for IKBKB, and −8.536 kcal/mol for PIK3CA. These results indicate potential interactions between baicalein and the predicted core targets.Proteins are shown as light-blue cartoon structures, baicalein is shown as green sticks, oxygen atoms are shown in red, nitrogen atoms are shown in blue, and blue dashed lines indicate hydrogen-bond interactions.

The molecular docking results showed that the binding energies between baicalein and all core target proteins were <−5.0 kcal/mol, indicating favorable binding affinities between baicalein and these targets. The docking conformations were visualized using PyMOL (Figure 9). The binding energies of baicalein with ABCC1, CD163, CYP1B1, IKBKB, and PIK3CA were −7.693, −7.958, −10.35, −5.717, and −8.536 kcal/mol, respectively.

#### 2.1.10. Molecular Dynamics Simulation

**Figure 10:**
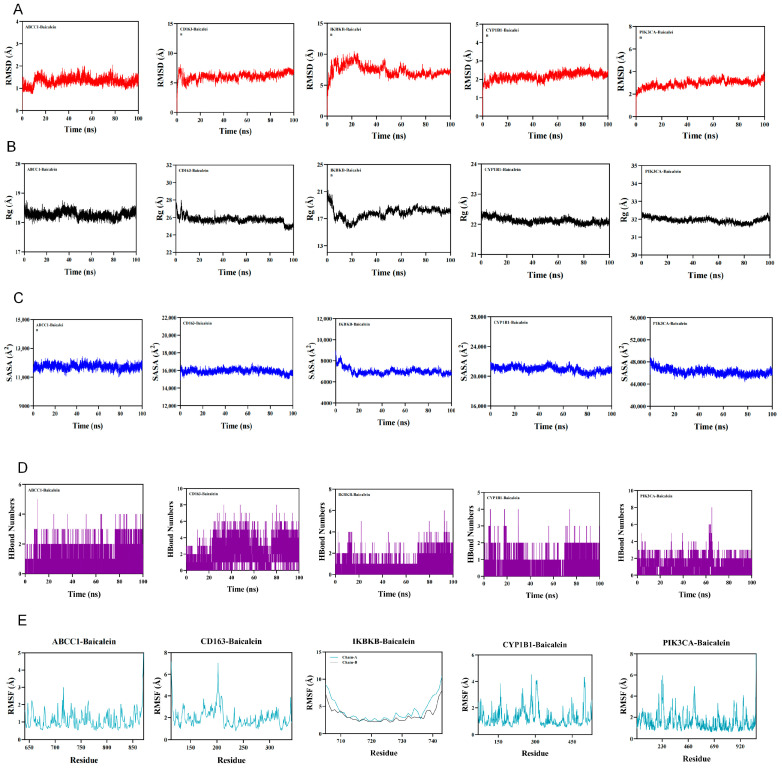
Molecular dynamics simulation of baicalein–target protein complexes. Molecular dynamics simulations were performed to evaluate the dynamic behaviors of the baicalein complexes with ABCC1, CD163, CYP1B1, IKBKB, and PIK3CA. (**A**) Root mean square deviation (RMSD) trajectories of the five protein–ligand complexes during the simulation. (**B**) Radius of gyration (Rg) plots showing the compactness of the complexes over time. (**C**) Solvent-accessible surface area (SASA) plots reflecting changes in protein surface exposure after ligand binding. (**D**) Time-dependent numbers of hydrogen bonds formed between baicalein and the target proteins. (**E**) Root mean square fluctuation (RMSF) plots showing residue-level flexibility in each complex. Overall, the ABCC1–, CYP1B1–, and PIK3CA–baicalein complexes showed relatively favorable dynamic stability, whereas the CD163– and IKBKB–baicalein complexes exhibited comparatively larger RMSD fluctuations, suggesting weaker structural support for stable binding.

The root mean square deviation (RMSD) is a useful indicator for evaluating the conformational stability of protein–ligand systems and the extent of deviation of atomic positions from their initial coordinates. Smaller deviations generally indicate greater conformational stability. Therefore, RMSD was used to assess the equilibration of the simulation systems. As shown in Figure 10A, the ABCC1–baicalein complex reached equilibrium after 20 ns and subsequently fluctuated around 1.5 Å. The CD163–baicalein complex reached equilibrium after 20 ns and fluctuated around 7 Å. The CYP1B1–baicalein complex reached equilibrium after 5 ns and then fluctuated around 2.3 Å. The IKBKB–baicalein complex reached equilibrium after 65 ns, with overall fluctuations remaining below 10 Å. The PIK3CA–baicalein complex reached equilibrium after 5 ns and ultimately fluctuated below 3.3 Å. These results suggest that the ABCC1–, CYP1B1–, and PIK3CA–baicalein complexes showed relatively favorable dynamic stability, whereas the higher RMSD values observed for the CD163– and IKBKB–baicalein complexes may reflect larger conformational rearrangements or less favorable dynamic stability. Therefore, the structural support for stable binding was weaker for CD163 and IKBKB than for the other three targets.

The radius of gyration (Rg) was used to describe overall structural changes and the compactness of protein structures. The ABCC1–baicalein and PIK3CA–baicalein complexes showed relatively stable fluctuations during the simulation process, indicating that no obvious expansion or contraction occurred in these systems. In contrast, the CD163–baicalein, CYP1B1–baicalein, and IKBKB–baicalein complexes exhibited slight fluctuations during the simulation, suggesting the occurrence of conformational changes during the dynamic process (Figure 10B).

The solvent-accessible surface area (SASA) was further calculated to evaluate changes in protein surface exposure after ligand binding (Figure 10C). The ABCC1–baicalein and PIK3CA–baicalein complexes exhibited no obvious SASA changes, indicating that ligand binding had only a limited effect on the overall protein structure. In contrast, the CD163–baicalein, CYP1B1–baicalein, and IKBKB–baicalein complexes showed slight fluctuations, suggesting that ligand binding altered the local binding microenvironment to a certain extent.

Hydrogen bonds play an important role in ligand–protein interactions. The number of hydrogen bonds formed between baicalein and the target proteins during the molecular dynamics process is shown in Figure 10D. For the ABCC1–baicalein complex, the number of hydrogen bonds ranged from 0 to 5, with approximately 2 hydrogen bonds present in most cases. For the CD163–baicalein complex, the number ranged from 0 to 8, with approximately 4 hydrogen bonds observed most of the time. For the CYP1B1–baicalein complex, the number ranged from 0 to 4, with approximately 2 hydrogen bonds in most cases. For the IKBKB–baicalein complex, the number ranged from 0 to 6, with approximately 2 hydrogen bonds observed most frequently. For the PIK3CA–baicalein complex, the number ranged from 0 to 8, with approximately 3 hydrogen bonds present in most cases. These results suggest that baicalein maintained hydrogen-bonding interactions with all five target proteins during the simulation process.

The root mean square fluctuation (RMSF) reflects the flexibility of amino acid residues in a protein. As shown in Figure 10E, the ABCC1–baicalein complex exhibited relatively low RMSF values, mostly below 2 Å. The CD163–baicalein and CYP1B1–baicalein complexes showed RMSF values mostly below 4 Å, the IKBKB–baicalein complex mostly below 5 Å, and the PIK3CA–baicalein complex mostly below 6 Å. These findings suggest that no marked residue-level instability was observed in the simulated complexes, although residue flexibility differed among targets.

Taken together, the molecular dynamics results suggest that ABCC1–baicalein, CYP1B1–baicalein, and PIK3CA–baicalein exhibited relatively more favorable dynamic behavior, whereas CD163–baicalein and IKBKB–baicalein showed weaker structural support for stable binding because of their comparatively higher RMSD values. Nevertheless, hydrogen-bonding interactions and residue-level fluctuations did not indicate complete instability in these two complexes. Therefore, the molecular dynamics analysis provides preliminary and differential structural support for the interactions between baicalein and the prioritized targets, rather than equally strong evidence for all five complexes.

#### 2.1.11. Virtual Knockout Results

**Figure 11:**
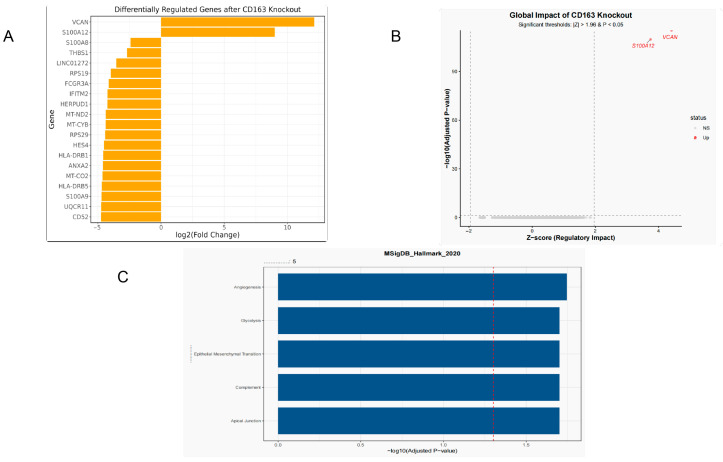
Virtual knockout analysis of CD163. A virtual knockout of CD163 was performed to explore its potential downstream regulatory effects. (**A**) Bar plot showing the top 20 differentially regulated genes after CD163 knockout. (**B**) Global impact plot showing the overall transcriptional perturbation induced by CD163 knockout; genes exceeding the significance thresholds are highlighted, including VCAN and S100A12. (**C**) Pathway enrichment analysis of the perturbed genes after CD163 knockout, showing that the altered genes were mainly enriched in pathways related to inflammation, immune regulation, and extracellular matrix remodeling. These results suggest that CD163 may participate in the regulation of downstream gene networks associated with COPD progression. The red dashed line represents the significance threshold of adjusted *p* = 0.05.

Following in silico knockout of CD163 (CD163 KO), the fold changes in the top 20 significantly differentially expressed genes indicated that these genes may be closely associated with disease progression under conditions of CD163 deficiency. The remaining genes (including the other 18 genes not specifically listed) showed progressively smaller magnitudes of change, and the overall distribution exhibited a pattern dominated by positive regulation (Figure 11A).

The global impact analysis further illustrated the effect of CD163 virtual knockout (CD163 KO) on overall gene expression regulation. The x-axis represents the Z-score, with a threshold of >1.96 indicating significant upregulation, while the y-axis represents −log10(adjusted *p*-value), with a threshold of >1.3 corresponding to a significance level of *p* < 0.05. Red dots represent genes that simultaneously met the significance thresholds for both Z-score and *p*-value, including VCAN and S100A12, which were classified as showing significant changes. Gray dots indicate genes with no significant change (NS) and are distributed below the threshold lines. This figure visually demonstrates the distribution of gene perturbations induced by CD163 knockout and highlights the significant regulatory effects of a small number of key genes (Figure 11B).

The KEGG pathway enrichment analysis of differentially expressed genes following in silico knockout of CD163 (CD163 KO) was performed using the Enrichr tool. The x-axis represents the enrichment score or −log10(*p*-value), while the y-axis lists the significantly enriched pathway terms. The length of each bar reflects the significance level of the corresponding pathway, and the color gradient may be used to distinguish different *p*-value ranges, with darker colors indicating higher significance. The enriched pathways were mainly associated with processes such as inflammatory responses, immune signaling, and extracellular matrix remodeling (for example, pathways potentially related to VCAN, as inferred from the context), suggesting that CD163 knockout affected these biological processes. This analysis further highlights the potential mechanistic role of CD163 in regulating downstream pathways (Figure 11C).

Through in silico knockout analysis based on scRNA-seq data, we revealed the critical regulatory role of CD163 in disease pathogenesis. The virtual knockout results suggested that CD163 may be associated with regulatory networks involved in inflammatory responses and the extracellular matrix. KEGG enrichment analysis of the perturbed genes after knockout further indicated that these genes may be involved in immune signaling and inflammation-related pathways. These findings provide computationally predicted evidence for the potential downstream network effects of CD163, although further experimental validation is still required.

### 2.2. In Vitro Cell Experiment Results

#### 2.2.1. Effect of Baicalein on the Viability of Inflammatory BEAS-2B Cells

**Figure 12:**
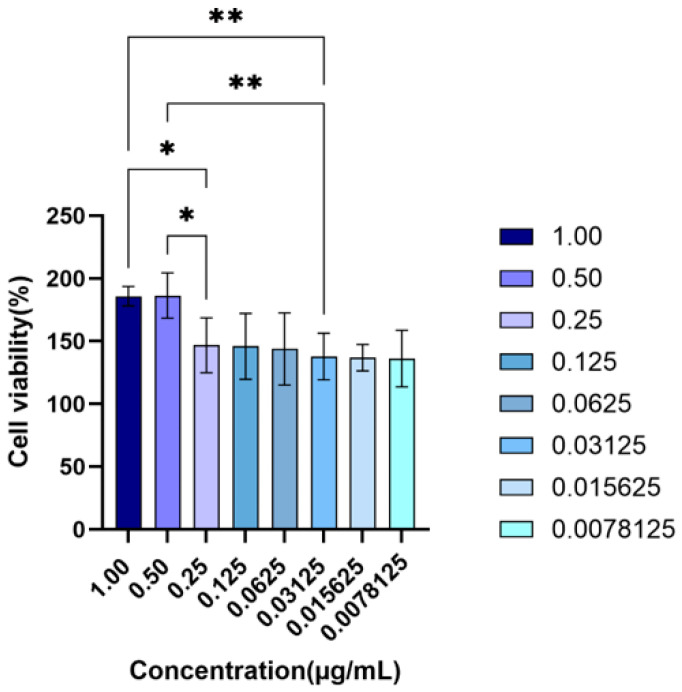
Effects of baicalein on the viability of LPS-stimulated BEAS-2B cells. Cell viability was evaluated using the CCK-8 assay after BEAS-2B cells were treated with different concentrations of baicalein for 24 h. Baicalein was tested at concentrations ranging from 1.00 to 0.0078125 μg/mL, and no obvious cytotoxicity was observed within this concentration range. The 1.00 and 0.50 μg/mL groups showed relatively higher cell viability and were selected as the high-dose and low-dose baicalein groups, respectively, for subsequent experiments. Data are presented as mean ± SD (*n* = 6). Statistical significance between the indicated groups is shown as * *p* < 0.05 and ** *p* < 0.01.

As shown in Figure 12, the effect of baicalein on the viability of LPS-stimulated BEAS-2B cells was evaluated using the CCK-8 assay. After 24 h of treatment, baicalein at concentrations of 1, 0.5, 0.25, 0.125, 0.0625, 0.03125, 0.015625, and 0.0078125 µg·mL^−1^ (corresponding to 3.70, 1.85, 0.93, 0.46, 0.23, 0.12, 0.06, and 0.03 µM, respectively) showed no obvious cytotoxicity and tended to improve cell viability within the tested concentration range. Selected pairwise comparisons further showed that cell viability in the 0.5 µg·mL^−1^ (1.85 µM) and 1 µg·mL^−1^ (3.70 µM) groups was significantly higher than that in the 0.25 µg·mL^−1^ (0.93 µM) group (*p* < 0.05), and was also significantly higher than that in the 0.03125 µg·mL^−1^ (0.06 µM) group (*p* < 0.01). Relatively stronger viability-promoting effects were therefore observed at 1 and 0.5 µg·mL^−1^ (1.85 µM), and these two concentrations were selected as the high- and low-dose groups for subsequent experiments.

#### 2.2.2. Effect of Baicalein on the Migration of Inflammatory BEAS-2B Cells

**Figure 13:**
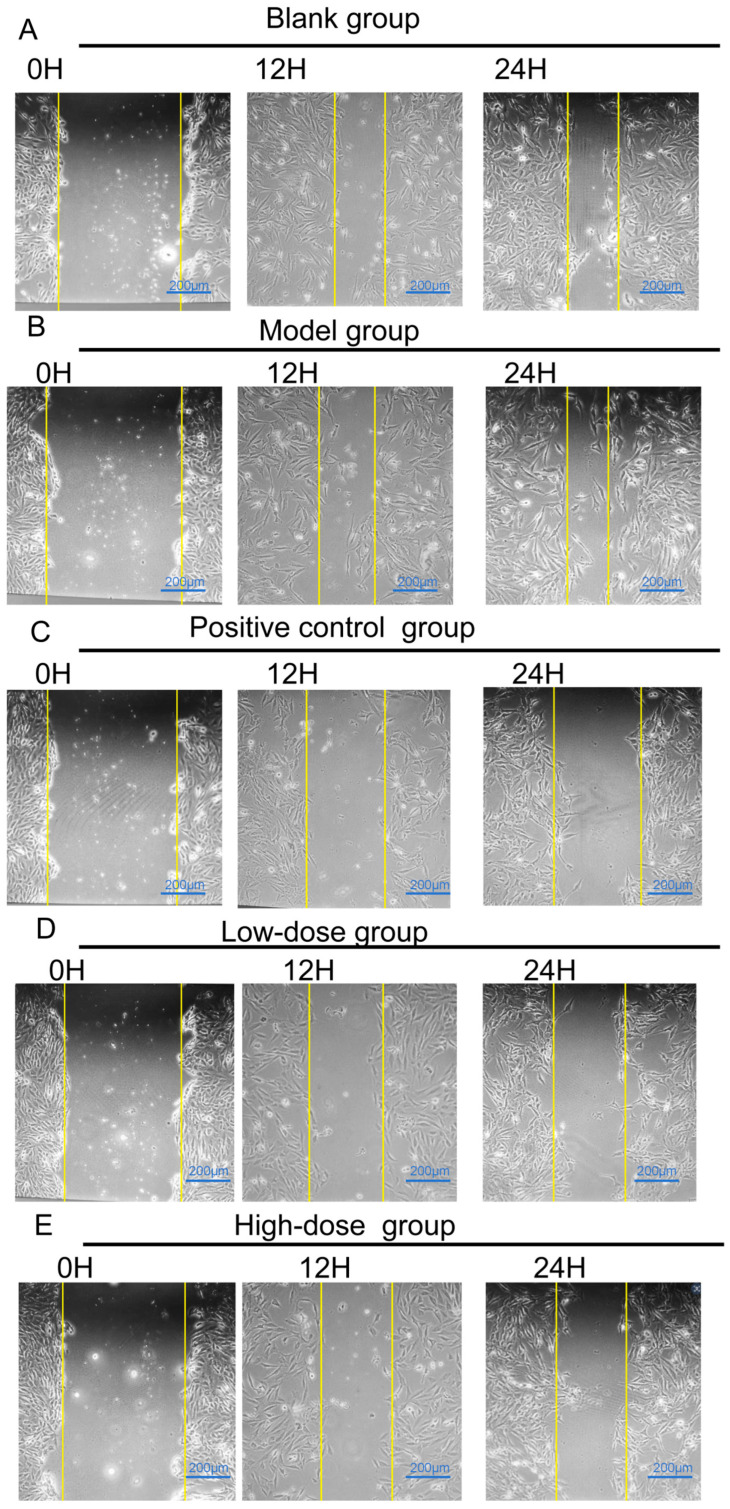
Effects of baicalein on BEAS-2B cell migration in an LPS-induced inflammatory model. Representative images from the wound-healing assay showing BEAS-2B cell migration at 0, 12, and 24 h after scratching. Cells were divided into the blank group, model group, positive control group, low-dose baicalein group, and high-dose baicalein group. (**A**) Blank group; (**B**) model group; (**C**) positive control group; (**D**) low-dose baicalein group; (**E**) high-dose baicalein group.The low- and high-dose baicalein groups were treated with 0.5 μg/mL and 1.0 μg/mL baicalein, respectively. The yellow lines indicate the wound boundaries at each time point. Compared with the model group, baicalein treatment delayed wound closure at 12 and 24 h, suggesting reduced migration-related activity in the LPS-induced BEAS-2B inflammatory model. Scale bar = 200 μm.

**Figure 14:**
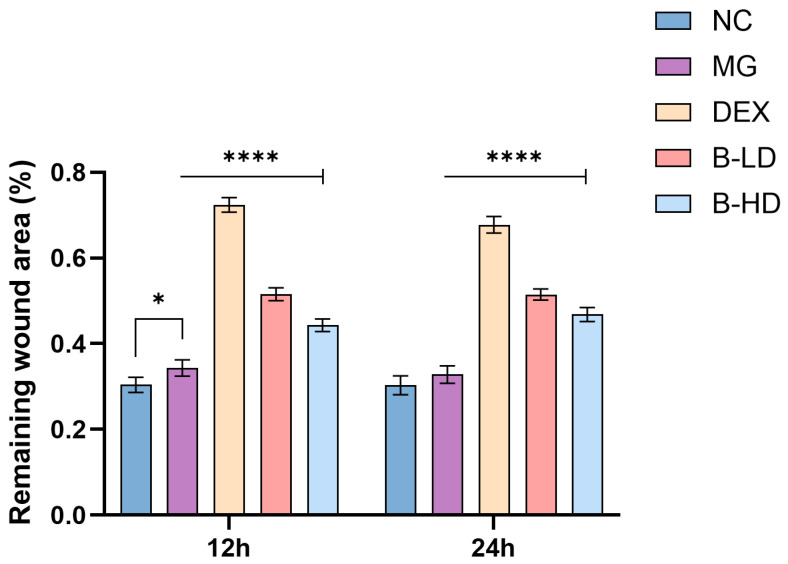
Effects of baicalein on the remaining wound area in an LPS-induced BEAS-2B inflammatory model. The remaining wound area was quantified at 12 and 24 h after scratching to evaluate the effect of baicalein on BEAS-2B cell migration under inflammatory conditions. Compared with the model group, both the low-dose and high-dose baicalein groups showed increased remaining wound area, indicating inhibited wound closure and reduced migration-related activity. The dexamethasone group showed the strongest inhibitory effect on wound closure. Data are presented as mean ± SD (*n* = 6). NC, normal control; MG, model group; DEX, dexamethasone group; B-LD, baicalein low-dose group; B-HD, baicalein high-dose group. Statistical significance between the indicated groups is shown as * *p* < 0.05 and **** *p* < 0.0001.

The wound-healing assay showed that baicalein treatment at 0.5 µg·mL^−1^ (1.85 µM) and 1 µg·mL^−1^ (3.70 µM) increased the remaining wound area of BEAS-2B cells at both 12 h and 24 h, indicating reduced lateral migratory capacity under inflammatory conditions. Compared with the model group, both baicalein-treated groups showed significant inhibitory effects on the migration-related phenotype at 12 h and 24 h. Specifically, the low-dose group showed highly significant differences at both 12 h and 24 h (*p* < 0.0001), whereas the high-dose group showed a significant effect at 12 h (*p* < 0.0001) and a highly significant effect at 24 h (*p* < 0.0001). These results suggest that baicalein suppressed the migration-related phenotype of BEAS-2B cells in the LPS-stimulated inflammatory model. See Figure 13 and Figure 14.

#### 2.2.3. Inhibitory Effect of Baicalein on Inflammatory Cytokines in Inflammatory BEAS-2B Cells

**Figure 15:**
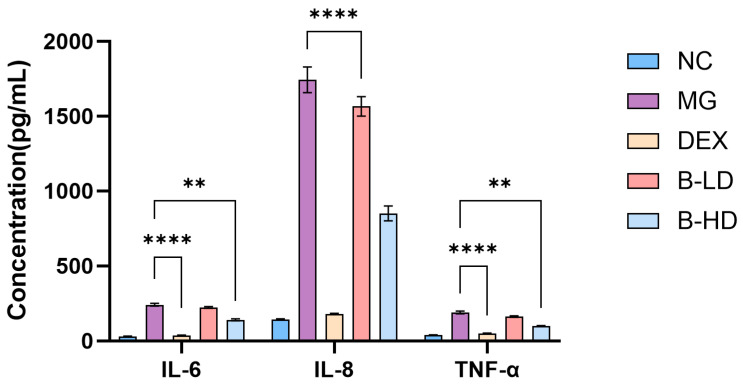
Effects of baicalein on inflammatory cytokine levels in an LPS-induced BEAS-2B inflammatory model. The levels of IL-6, IL-8, and TNF-α in the culture supernatants were determined by ELISA to evaluate the anti-inflammatory effect of baicalein. Compared with the NC group, the MG group showed markedly increased levels of IL-6, IL-8, and TNF-α, indicating successful induction of an inflammatory response. Treatment with dexamethasone and high-dose baicalein reduced the levels of all three cytokines, whereas low-dose baicalein mainly reduced IL-8, with weaker effects on IL-6 and TNF-α. Data are presented as mean ± SD (*n* = 6). NC, normal control; MG, model group; DEX, dexamethasone group; B-LD, baicalein low-dose group; B-HD, baicalein high-dose group. Statistical significance between the indicated groups is shown as ** *p* < 0.01 and **** *p* < 0.0001.

The levels of the inflammatory cytokines IL-6, IL-8, and TNF-α were measured using ELISA kits. As shown in Figure 15, compared with the blank group, the levels of the pro-inflammatory cytokines IL-8, IL-6, and TNF-α were significantly increased in the model group (*p* < 0.0001). Compared with the blank group, the positive control group, high-dose group all showed reduced levels of IL-6, IL-8, and TNF-α to varying degrees (*p* < 0.01). The low-dose group showed a significant reduction in IL-8 (*p* < 0.0001), whereas the levels of IL-6 and TNF-α were reduced to some extent but did not differ significantly from those in the model group (*p* > 0.05).

Among them, the positive control group showed the greatest reduction, followed by the high-dose group, whereas the low-dose group showed the smallest reduction. These findings indicate that baicalein can reduce inflammatory cytokine levels in a dose-dependent manner.

#### 2.2.4. Effect of Baicalein on the Expression of Core Target-Related and Inflammation-Related Genes in Inflammatory BEAS-2B Cells

**Figure 16:**
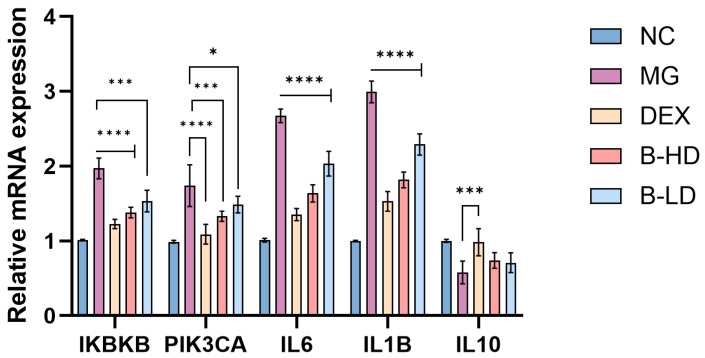
Effects of baicalein on the mRNA expression of core target-related and inflammation-related genes in an LPS-induced BEAS-2B inflammatory model. The relative mRNA expression levels of IKBKB, PIK3CA, IL6, IL1B, and IL10 were measured by RT-qPCR to evaluate the regulatory effects of baicalein on core target-related and inflammatory genes. Compared with the NC group, the MG group showed increased expression of IKBKB, PIK3CA, IL6, and IL1B, together with decreased IL10 expression, indicating successful induction of an inflammatory response. Dexamethasone and baicalein treatment reduced the expression of IKBKB, PIK3CA, IL6, and IL1B to varying degrees, with the high-dose baicalein group generally showing a stronger inhibitory effect than the low-dose group. IL10 expression showed a partial recovery trend after baicalein treatment. Data are presented as mean ± SD (*n* = 6). NC, normal control; MG, model group; DEX, dexamethasone group; B-HD, baicalein high-dose group; B-LD, baicalein low-dose group. Statistical significance between the indicated groups is shown as * *p* < 0.05, *** *p* < 0.001, and **** *p* < 0.0001.

BEAS-2B cells were assigned to the normal control group (NC), model group (MG), dexamethasone group (DEX), baicalein high-dose group (B-HD), and baicalein low-dose group (B-LD). Following treatment, total RNA was isolated, and RT-qPCR was performed to determine the mRNA expression levels of IKBKB, PIK3CA, IL1B, IL6, and IL10. As shown in Figure 16, LPS stimulation significantly increased the expression of IKBKB, PIK3CA, IL1B, and IL6 while decreasing IL10 expression in BEAS-2B cells compared with the NC group (*p* < 0.05), confirming the successful establishment of the inflammatory model. Compared with the MG group, DEX significantly reversed these changes, as reflected by the downregulation of IKBKB, PIK3CA, IL1B, and IL6 and the upregulation of IL10 (*p* < 0.05). Baicalein intervention also reduced the expression of IKBKB, PIK3CA, IL1B, and IL6, with the B-HD group generally showing a greater effect than the B-LD group. In contrast, IL10 expression showed a recovery trend after baicalein treatment, although no statistically significant difference was observed relative to the MG group. Collectively, these findings indicate that baicalein alleviates LPS-induced inflammatory responses at the epithelial-cell level and modulates selected core target-related genes, thereby strengthening the mechanistic coherence between the computational analyses and the in vitro validation.

## 3. Discussion

Chronic obstructive pulmonary disease (COPD) is a biologically heterogeneous respiratory disorder driven by persistent inflammation, immune dysregulation, abnormal tissue repair, and structural remodeling. Rather than being governed by a single inflammatory axis, COPD involves a broader network of immune- and inflammation-related pathways, including PI3K-Akt, NF-κB, TNF, and JAK-STAT signaling [4]. In addition, the disease is increasingly recognized as a multicellular process involving extensive crosstalk between immune cells and structural cells. In particular, macrophages and airway epithelial cells jointly contribute to inflammatory amplification, epithelial injury, mucus dysregulation, and pathological remodeling. Against this background, the present study adopted an integrative, multilayered strategy to prioritize biologically plausible targets and candidate mechanistic directions associated with baicalein in COPD, rather than to provide definitive mechanistic proof.

As shown in Figure 16, at the level of target integration and functional annotation, the present study predicted the potential targets of baicalein using CTD, SEA, and SwissTargetPrediction, and intersected them with COPD-related genes collected from GeneCards and OMIM. Functional enrichment analysis suggested that the overlapping targets were mainly associated with inflammation-, immunity-, and migration-related pathways. These findings provided an initial systems-level framework for subsequent target prioritization. However, because broad disease-gene resources may introduce noise owing to variable levels of supporting evidence and overlap with other disease contexts, the downstream targets identified here should be interpreted as prioritized candidate targets rather than definitive COPD-specific genes.

At the transcriptomic and machine learning levels, five genes, namely ABCC1, CD163, CYP1B1, IKBKB, and PIK3CA, were prioritized as core candidate genes through integrated analysis. Immune infiltration and single-cell transcriptomic analyses further suggested that macrophage-associated immune regulation may represent an important mechanistic direction in COPD. This observation is biologically plausible, given the well-established role of macrophages in inflammatory amplification and tissue injury in COPD. At the same time, the airway epithelium is now recognized as an immunologically active interface that senses environmental injury and communicates with resident and recruited immune cells [18]. Moreover, macrophage–epithelial crosstalk has been directly demonstrated in a cigarette-smoke-related context, in which macrophages influenced BEAS-2B epithelial phenotypes under smoke-related stimulation [19]. Therefore, the current computational results should be interpreted as highlighting a macrophage-associated immune axis within a broader multicellular disease context.

At the genetic level, formal MR follow-up was ultimately conducted only for CD163, and the results provided supportive genetic evidence for prioritizing this target in COPD. However, the MR findings should be interpreted cautiously. Although the instrumental variables showed adequate strength and no significant pleiotropy or heterogeneity was detected, formal bidirectional MR, MR-PRESSO, and colocalization analyses were not incorporated. Therefore, the MR results are more appropriately viewed as supportive evidence for target prioritization rather than definitive causal proof.

At the structural level, molecular docking suggested that baicalein may interact favorably with several prioritized targets, and molecular dynamics simulation provided additional dynamic information on target–compound complexes. However, the structural evidence was not equally strong across all targets. In particular, the direct structural support for IKBKB and CD163 was weaker than that for several other targets, and these two candidates were retained primarily because of their transcriptomic, cellular, and biological relevance rather than because they showed the strongest docking or molecular dynamics behavior. Thus, the structural analyses in the present study should be interpreted as preliminary and supportive rather than conclusive.

To further estimate the possible systems-level effects of key nodes, this study also introduced virtual knockout analysis. The results suggested that virtual knockout of CD163 perturbed a subset of downstream genes and pathways related to inflammation and immune regulation. Nevertheless, this analysis was based on network inference and in silico perturbation rather than true biological knockout. It therefore provides supplementary evidence regarding potential downstream network sensitivity, but cannot replace actual genetivalidation. The present study used LPS-stimulated BEAS-2B cells as an epithelial inflammatory model. This model was not intended to serve as a surrogate for macrophages, but rather to assess whether baicalein could suppress airway epithelial inflammatory output, which is also an important component of COPD pathology. Previous studies have likewise shown that baicalein exerts anti-inflammatory effects in pulmonary inflammatory settings. For example, Gao et al. reported that baicalein alleviated LPS-induced acute lung injury and suppressed inflammatory responses and pyroptosis, with PI3K-Akt, TNF, and NF-κB identified as major pathway-level signals [13]. In this respect, the present study is broadly consistent with prior evidence showing that baicalein is anti-inflammatory in lung injury-related contexts. However, the current work extends this line of research by emphasizing COPD-oriented target prioritization, immune-cell context, and candidate mechanistic direction, rather than simply re-identifying broad inflammatory pathways.

In the present epithelial model, baicalein improved CCK-8 metabolic activity in LPS-stimulated BEAS-2B cells and reduced inflammatory cytokine secretion, including IL-6, IL-8, and TNF-α, suggesting attenuation of inflammatory output. In addition, the wound-healing assay showed that baicalein increased the remaining wound area of inflammatory BEAS-2B cells, indicating suppression of the migration-related phenotype under the present experimental conditions. Furthermore, the newly added RT-qPCR results showed that baicalein downregulated IKBKB, PIK3CA, IL1B, and IL6, while partially restoring IL10 expression. These findings strengthen the consistency between the computational predictions and the epithelial-cell experiments, although they still represent epithelial-level supportive validation rather than direct confirmation of the macrophage-centered mechanism.

Several limitations should be acknowledged. First, the present study relied heavily on public databases and integrated transcriptomic datasets, and the initial disease-gene set may still contain noise despite cross-database integration and batch correction. Second, only one single-cell dataset was used, and the CIBERSORT filtering process could not be fully reconstructed at the sample-count level from the archived files. Third, the computational layers, including MR, docking, molecular dynamics simulation, and virtual knockout analysis, are inherently predictive and supportive rather than definitive, and some targets—especially CD163 and IKBKB—showed weaker structural support than others. Fourth, the LPS-stimulated BEAS-2B model reflects epithelial inflammatory responses but does not fully recapitulate the complexity of COPD, for which cigarette-smoke-related epithelial models, macrophage-based systems, and in vivo models would be more informative. Finally, the in vitro concentrations of baicalein used here should be interpreted as exploratory exposures for mechanistic evaluation rather than direct clinical equivalents, and their translational relevance, optimal delivery route, non-inflammatory cytotoxicity window, and in vivo feasibility remain to be established [20].

**Figure 17:**
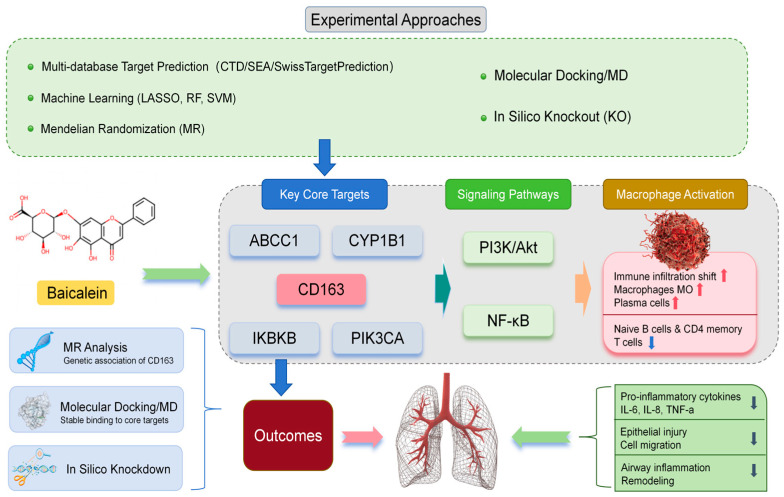
The potential molecular mechanism by which baicalein intervenes in COPD may involve targeting core molecules such as ABCC1, CD163, CYP1B1, IKBKB, and PIK3CA, thereby regulating inflammation- and immune-related signaling pathways, including PI3K/Akt and NF-κB, modulating the macrophage-associated immune microenvironment, and ultimately suppressing the release of pro-inflammatory cytokines while alleviating airway epithelial injury and the trend toward abnormal cell migration. Arrows indicate the direction of the proposed analytical workflow and potential mechanistic relationships.

Overall, the present study supports the view that baicalein may exert anti-inflammatory effects in COPD through a multi-target, immune-associated framework, with macrophage-related regulation and CD163 emerging as particularly noteworthy candidate directions. More importantly, the biological contribution of this study lies not in re-establishing general pathway-level associations alone, but in narrowing candidate mechanisms and targets within the COPD context through an integrative, hypothesis-generating strategy. Future work should therefore focus on macrophage-based validation, in vivo confirmation, and more direct target-level intervention experiments to determine whether the prioritized mechanisms identified here can be translated into biologically and clinically meaningful evidence (Figure 17).

## 4. Materials and Methods

### 4.1. Materials and Instruments

BEAS-2B cells (human normal bronchial epithelial cells) were purchased from Wuhan Sunen Biotechnology Co., Ltd. (Wuhan, China). Baicalein (catalog no. B20571-20 mg; purity: HPLC ≥ 98%) and DMEM medium (catalog no. R28175-599 mL) were purchased from Shanghai Yuanye Biotechnology Co., Ltd. (Shanghai, China). Fetal bovine serum (catalog no. BS-1105) was purchased from Inner Mongolia Opcel Biotechnology Co., Ltd. (Hohhot, China). The CCK-8 kit (catalog no. LV08-500) was purchased from Invigentech, Inc. (Irvine, CA, USA). LPS (catalog no. S11060-10 mg) was purchased from Shanghai Yuanye Biotechnology Co., Ltd. Dexamethasone (catalog no. S17003-1 g) was purchased from Shanghai Yuanye Biotechnology Co., Ltd. Human interleukin-8 (IL-8) ELISA kit (catalog no. JL19291) was purchased from Shanghai Jianglai Industrial Co., Ltd. (Shanghai, China). Human interleukin-6 (IL-6) ELISA kit (catalog no. JL14113) was purchased from Shanghai Jianglai Industrial Co., Ltd. (Shanghai, China). Human tumor necrosis factor-α (TNF-α) ELISA kit (catalog no. JL10208) was purchased from Shanghai Jianglai Industrial Co., Ltd. (Shanghai, China). TRIzol reagent was purchased from Invitrogen; Thermo Fisher Scientific (Waltham, MA, USA). Forward and reverse primers were synthesized by Sangon Biotech Co., Ltd. (Shanghai, China). NovoScript Plus All-in-one 1st Strand cDNA Synthesis SuperMix (gDNA Purge), and NovoStart SYBR qPCR SuperMix Plus kit (Novoprotein Scientific Inc., Suzhou, China).

The instruments used in this study included a microplate reader (SpectraMax Paradigm, Molecular Devices, San Jose, CA, USA), a CO_2_ cell incubator (Model 4111, Thermo Fisher Scientific, Waltham, MA, USA), a refrigerated centrifuge (Smart R17 Plus, Hanil Scientific Inc., Gimpo, Republic of Korea), a real-time fluorescence quantitative PCR system (Thermo Fisher Scientific, USA), and a biosafety cabinet (Thermo Fisher Scientific, USA).

### 4.2. Experimental Methods

#### 4.2.1. Construction of the Baicalein–COPD Interaction Network and Multi-Omics Integrative Analysis

Baicalein is a natural flavonoid with well-documented anti-inflammatory and immunomodulatory properties, and its predicted targets substantially overlap with molecular networks associated with COPD. Therefore, baicalein was selected as the core compound of this study, and a baicalein–COPD interaction network was constructed. By integrating multi-omics data with multiple analytical strategies, we systematically investigated its potential molecular mechanisms.

##### Transcriptomic Data Acquisition and Preprocessing

In this study, gene expression profile data for chronic obstructive pulmonary disease (COPD) were obtained from the Gene Expression Omnibus (GEO, https://www.ncbi.nlm.nih.gov/geo/ (accessed on 17 February 2026)) database. The search term used was “chronic obstructive pulmonary disease”, with the following inclusion criteria: entry type = “Dataset”, organism = Homo sapiens, and study type = “Expression profiling by high-throughput sequencing”. Ultimately, four datasets, namely GSE20257, GSE42057, GSE76925, and GSE130928, were included. After batch-effect correction using the sva (version 3.59.0) R package, the datasets were merged, resulting in a combined cohort of 260 normal samples and 250 COPD tissue samples.

##### Prediction of Baicalein Targets

The potential targets of baicalein were predicted using the SwissTargetPrediction (http://swisstargetprediction.ch/ (accessed on 17 February 2026)), Comparative Toxicogenomics Database (CTD, https://ctdbase.org/ (accessed on 17 February 2026)), and Similarity Ensemble Approach (SEA, https://sea.bkslab.org/ (accessed on 17 February 2026)) databases.

##### Acquisition of COPD-Related Targets and Identification of Overlapping Targets

COPD-related targets were collected from the GeneCards (https://www.genecards.org/ (accessed on 18 February 2026)) and OMIM (https://omim.org/ (accessed on 18 February 2026)) databases using “chronic obstructive pulmonary disease” as the search term. After merging the results from the two databases and removing duplicate entries, a candidate COPD-related target set was obtained for downstream analysis. Because broad disease-gene resources may include entries supported by different levels of evidence and targets overlapping with other disease contexts, the resulting set was used as an initial candidate pool rather than as a definitive collection of COPD-specific targets.

##### Identification of Overlapping Targets

First, the predicted baicalein targets obtained from the above three databases were intersected to generate the final set of drug-predicted targets. Subsequently, this set was intersected with the COPD-related target set to identify the potential overlapping targets through which baicalein may exert therapeutic effects against COPD. This procedure was performed using the Venn package in R (version 1.12).

##### Functional Enrichment Analysis

To elucidate the biological functions and related pathways of the overlapping targets, Gene Ontology (GO) enrichment analysis and Kyoto Encyclopedia of Genes and Genomes (KEGG) pathway analysis were performed. The GO analysis comprised three categories, including Biological Process (BP), Cellular Component (CC), and Molecular Function (MF), with the aim of systematically revealing the biological processes and potential signaling pathways associated with the overlapping targets.

##### Machine Learning-Based Screening of Feature Genes

To further identify key feature genes from the overlapping targets, three machine learning algorithms were employed in this study, including least absolute shrinkage and selection operator (LASSO) regression, support vector machine (SVM), and random forest (RF). In the LASSO analysis, the glmnet (version 5.0) R package was used with a penalty parameter, and 10-fold cross-validation was performed to identify key variables from high-dimensional data. Random forest is an ensemble learning algorithm that improves predictive accuracy and robustness by constructing multiple decision trees and integrating their classification results, with the final output determined by majority voting. Support vector machine achieves sample classification by constructing an optimal separating hyperplane in a high-dimensional feature space and is particularly suitable for handling complex data. Finally, the genes jointly identified by the three methods were intersected using the ggvenn (version 0.1.10) R package and were defined as the core genes.

##### Differential Analysis and Visualization of Core Genes

The expression levels of the core genes in the normal and COPD groups were extracted, and differential expression analysis between groups was performed using the limma (version 3.58.1), pheatmap (version 1.0.12), and ggpubr (version 0.6.0) R packages. The results were presented as box plots and heatmaps, and genes with *p* < 0.05 were defined as significantly differentially expressed core genes. In addition, chromosomal localization of the core genes was performed using the circlize (version 0.4.15) R package. Furthermore, based on the expression data of the core genes in COPD samples, Spearman correlation coefficients among these genes were calculated using the corrplot (version 0.95) and circlize (version 0.4.15) R packages, and the results were visualized by circular plots and heatmaps.

##### Immune Infiltration Analysis

In this study, the CIBERSORT deconvolution algorithm was applied to estimate the proportions of 22 immune cell types in mixed samples based on the known gene expression signatures of immune cell subsets. After 1000 permutations, samples with a CIBERSORT deconvolution *p* < 0.05 were retained for subsequent immune infiltration analysis, and the immune cell composition of each sample was visualized using bar plots. The ggpubr (version 0.6.0) and reshape2 (version 1.4.5) R packages were used to compare and visualize differences in immune cell proportions between the normal and COPD groups. Finally, Spearman correlation analysis was performed to evaluate the associations between core gene expression and the infiltration levels of specific immune cells, and the results were presented as a heatmap.

##### SHAP Analysis

Based on the identified core genes, ten machine learning models were constructed, including ridge least squares (RLS), random forest (RF), decision tree (DTS), support vector machine (SVM), logistic regression, k-nearest neighbor (KNN), extreme gradient boosting (XGBoost), gradient boosting machine (GBM), neural network, and generalized linear model boosting (GlmBoost). Model performance was evaluated from multiple dimensions, including the receiver operating characteristic (ROC) curve, specificity, sensitivity, and accuracy, with the ROC curve used as the primary evaluation metric. The model with the best performance was selected as the final predictive model. To enhance model interpretability, Shapley Additive exPlanations (SHAP) analysis was further performed to quantify the contribution of each core gene feature to the prediction results, thereby revealing the underlying decision-making mechanism of the model.

##### Single-Gene GSVA

Gene Set Variation Analysis (GSVA) is a non-parametric and unsupervised method for gene set enrichment analysis. In this study, the gene set “c2.cp.kegg.symbols.gmt” was downloaded from the GSEA database (http://www.gsea-msigdb.org/ (accessed on 19 February 2026)), and the “GSVA” (version 2.6.1) and “limma” (version 3.58.1) R packages were used to analyze the enrichment of the core gene set in KEGG pathways. A significance threshold of *p* < 0.05 was applied.

##### Single-Cell Data Analysis

The single-cell RNA-seq dataset GSE167295 was downloaded from the Gene Expression Omnibus (GEO) database. According to the GEO record, this dataset contains 29,961 single cells derived from peripheral lung parenchymal tissue of three patients with severe COPD and three nonsmoking subjects without underlying lung disease. Based on this dataset, rigorous quality control was first performed as follows: (1) only cells expressing at least 200 genes, with these genes detected in at least 3 cells, were retained; and (2) cells with mitochondrial and ribosomal gene proportions greater than 20% were excluded. Gene expression was normalized using the LogNormalize method, and batch effects were corrected using the harmony R package (version 2.0.2). Appropriate principal components (PCs) were selected according to the ElbowPlot results, after which cell clustering was performed using the FindClusters function in the Seurat package with the resolution set to 1.2. The clustering results were then visualized by dimensionality reduction using Uniform Manifold Approximation and Projection (UMAP). Cell subpopulations were annotated using the SingleR tool. Furthermore, the expression distribution of the core genes across different cell clusters was visualized using FeaturePlot, and the enrichment scores of the core gene set were calculated using the UCell (version 2.16.0) and irGSEA (version 3.2.2) R packages, followed by density distribution analysis. In the present study, the single-cell dataset was used as a complementary resource for cellular localization of the prioritized core genes, rather than as the primary discovery dataset.

##### Plasma Protein Mendelian Randomization

To further investigate the genetic support for prioritized core targets in COPD, a two-sample Mendelian randomization (MR) analysis was performed using the TwoSampleMR R package (version 0.5.8). Among the five prioritized core genes, formal protein-level MR analysis was ultimately conducted only for CD163, because it emerged as the most biologically notable candidate in the integrated analysis, particularly in relation to immune-cell localization and macrophage-associated relevance, and suitable instrumental-variable data were available for downstream MR evaluation. Plasma protein data for CD163 were obtained from the deCODE Health study, and the outcome data were derived from the IEU OpenGWAS database (ID: ebi-a-GCST90018807). Single-nucleotide polymorphisms (SNPs) were used as instrumental variables (IVs) to assess the association between genetically predicted CD163 plasma protein levels and COPD risk. A significance threshold of *p* < 5 × 10^−8^ was applied for IV selection, followed by linkage disequilibrium pruning (r^2^ = 0.001, kb = 10,000) to ensure instrument independence. In this study, the inverse variance weighted (IVW) method was used as the primary MR approach, and an IVW *p* < 0.05 was considered statistically significant. In addition, Cochran’s Q statistic was used to assess heterogeneity, while MR-Egger regression was used to detect directional horizontal pleiotropy. The F statistic was also calculated to evaluate instrument strength, with F > 10 indicating a limited risk of weak instrument bias. Leave-one-out sensitivity analysis was further performed to evaluate whether the observed association was driven by a single SNP. By using genetic variants as IVs, MR can provide supportive evidence regarding the relationship between exposure and outcome while reducing the influence of confounding factors.

##### Molecular Docking of Baicalein

The 3D molecular structure of baicalein was downloaded from PubChem (https://pubchem.ncbi.nlm.nih.gov/ (accessed on 19 February 2026)). The protein structures of the core targets were obtained from the Protein Data Bank (PDB, https://www.rcsb.org/ (accessed on 19 February 2026)). PyMOL (version 2.6) was used for structure preprocessing, including removal of water molecules and other redundant ligands, as well as hydrogenation. Binding pockets were predicted using DeepSite (https://www.playmolecule.org/deepsite/ (accessed on 19 February 2026)), and semi-flexible molecular docking was performed using AutoDock Tools (version 1.5.6). The docking results were then analyzed using the PLIP website (https://plip-tool.biotec.tu-dresden.de/plip-web/plip/index (accessed on 20 February 2026)), and the analyzed docking results were imported into PyMOL for visualization.

##### Molecular Dynamics Simulation

In this study, molecular dynamics (MD) simulations were performed using GROMACS 2022, and the force field parameters were generated with the pdb2gmx tool in GROMACS. For the ligand, topology files based on the GAFF2 force field were generated using sobtop_1.0 (dev3.1) according to the ligand structure, and ligand charges were assigned using the RESP method to ensure that the charge distribution was consistent with its physicochemical properties. For the receptor proteins, the AMBER14SB force field was applied. During the simulation, the system was solvated using the TIP3P water model in a cubic water box with a 1 nm buffer distance to ensure adequate solvation and electrical neutrality. To further neutralize the system, Na^+^ and Cl^−^ ions were added using the gmx genion tool in GROMACS, and the ionic concentration was set to 0.15 M NaCl to mimic conventional physiological ionic strength under simulation conditions. Long-range electrostatic interactions were treated using the particle mesh Ewald (PME) method, with a cutoff distance of 1 nm. The force field parameters and PME-related settings were optimized in accordance with GROMACS specifications. Bond constraints were handled using the LINCS algorithm. Before the molecular dynamics simulation, the system underwent an energy minimization procedure. Energy minimization consisted of 3000 steps of steepest descent optimization, followed by 2000 steps of conjugate gradient optimization. The optimization process was carried out in three stages: first, the solute was restrained and the water molecules were energy-minimized; second, the counterions were restrained and energy minimization was performed; finally, the entire system underwent unrestrained energy minimization. During the simulation, the temperature was maintained at 310 K using the Nosé–Hoover thermostat, while the pressure was maintained at 1 bar using the Parrinello–Rahman barostat. The simulation time was set to 100 ns and conducted under the NPT (constant pressure and constant temperature) ensemble, with an integration time step of 2 fs. During the simulation, the GROMACS tools gmx rmsd, gmx rmsf, gmx hbond, gmx gyrate, and gmx sasa were used to calculate the root mean square deviation (RMSD), root mean square fluctuation (RMSF), hydrogen bonds (HBonds), radius of gyration (Rg), and solvent-accessible surface area (SASA), respectively, in order to analyze the stability, structural changes, and solvent effects of the system.

##### Virtual Knockout Analysis

To further investigate the potential role of the target gene in disease pathogenesis, a virtual knockout (KO) analysis was performed using the machine learning workflow implemented in the scTenifoldKnk (version 1.0.3) R package based on scRNA-seq data. The gene–cell expression matrix from the single-cell dataset was used as the input, and the target gene was virtually knocked out. scTenifoldKnk constructed two gene regulatory networks (GRNs): one representing the original dataset and the other generated after computational deletion of the target gene. Differentially regulated genes between the two networks were then compared. The perturbed genes were ranked according to fold-change values. This approach enabled the identification of genes affected by the virtual knockout of the target gene. Functional enrichment analysis was subsequently performed on the resulting differentially expressed genes to evaluate the potential biological functional changes associated with the virtual knockout of the target gene.

#### 4.2.2. In Vitro Cell Experiments

##### Cell Culture

BEAS-2B cells were cultured in DMEM medium in a humidified incubator at 37 °C with 5% CO_2_. Cells in the logarithmic growth phase were selected for subsequent experiments.

##### CCK-8 Assay for Cell Viability

BEAS-2B cells in the logarithmic growth phase were seeded into 96-well plates at a density of 5 × 10^3^ cells per well and cultured for 24 h. An inflammatory model was then induced with 1 µg·mL^−1^ LPS, with reference to previous studies using LPS-stimulated BEAS-2B cells as an in vitro airway inflammatory model [21]. After 24 h, baicalein was added at different concentrations of 1, 0.5, 0.25, 0.125, 0.0625, 0.03125, 0.015625, and 0.0078125 µg·mL^−1^ (corresponding to 3.70, 1.85, 0.93, 0.46, 0.23, 0.12, 0.06, and 0.03 µM, respectively) and incubated for another 24 h. Subsequently, CCK-8 solution was added, and the cells were incubated in the dark for 2 h. The absorbance value at 450 nm was measured, and cell viability was calculated accordingly.

##### Wound-Healing Assay for Cell Migration

BEAS-2B cells in the logarithmic growth phase were seeded into 6-well plates at a density of 2 × 10^5^ cells per well and cultured for 24 h until the cell confluence reached approximately 90%. The cells were then assigned to the normal control group (NC), model group (MG), dexamethasone group (DEX), baicalein low-dose group (B-LD), and baicalein high-dose group (B-HD). The NC group received vehicle treatment only, whereas the other groups were stimulated with 1 µg·mL^−1^ LPS to establish the inflammatory model. After that, a uniform scratch was made across the cell monolayer using a sterile pipette tip. The DEX group received dexamethasone treatment, while the B-LD and B-HD groups were treated with baicalein at 0.5 µg·mL^−1^ (1.85 µM) and 1 µg·mL^−1^ (3.70 µM), respectively. Images were captured at 12 and 24 h during incubation, and the remaining wound area was quantified to evaluate cell migration.

##### Determination of Inflammatory Cytokine Levels Using ELISA Kits

BEAS-2B cells in the logarithmic growth phase were seeded into 6-well plates at a density of 2 × 10^5^ cells per well and cultured for 24 h until the cell confluence reached approximately 90%. An inflammatory model was then induced with 1 µg·mL^−1^ LPS. The experimental groups in this assay were consistent with those described in Section Wound-Healing Assay for Cell Migration, including the blank group, model group, baicalein low-dose group, baicalein high-dose group, and positive control group. After the corresponding treatments had been administered for another 24 h, the cell culture supernatants were collected for the determination of inflammatory cytokine levels.

##### RT-qPCR Analysis of Related Gene Expression

BEAS-2B cells in the logarithmic growth phase were seeded into 6-well plates at a density of 3 × 10^5^ cells/well. After cell attachment, the experimental groups and treatment procedures were performed as described in Section Wound-Healing Assay for Cell Migration. Following treatment, total RNA was extracted from each group using TRIzol reagent, and the RNA concentration and purity were determined. RNA was then reverse-transcribed into cDNA according to the manufacturer’s instructions for the reverse transcription kit, followed by amplification using an RT-qPCR kit. Referring to the method of Xiao X et al. [22], with slight modifications, ACTB was used as the internal reference gene, and the relative expression levels of IKBKB, PIK3CA, IL6, IL1B, and IL10 were calculated using the 2^−ΔΔCt^ method. Primer sequences are listed in Table 1. The PCR cycling conditions were as follows: pre-denaturation at 95 °C for 2 min, followed by 40 cycles of 95 °C for 10 s and 60 °C for 30 s. A melting curve analysis was performed after amplification to verify the specificity of the products.

### 4.3. Statistical Analysis

Data are presented as mean ± SD. Statistical analyses were performed using GraphPad Prism 10.6. One-way ANOVA or two-way ANOVA was used as appropriate, followed by the corresponding multiple-comparisons test. A value of *p* < 0.05 was considered statistically significant. Statistical significance is indicated as follows: * *p* < 0.05, ** *p* < 0.01, *** *p* < 0.001, and **** *p* < 0.0001.Exact *p*-values for key comparisons are reported in the Results section where appropriate.

## 5. Conclusions

Taken together, the present study suggests that baicalein may exert anti-inflammatory effects in COPD through a multi-target, immune-associated framework. Specifically, ABCC1, CD163, CYP1B1, IKBKB, and PIK3CA were prioritized as noteworthy candidate targets, and macrophage-associated immune regulation emerged as a potentially important mechanistic direction. Baicalein may be associated with inflammation- and immune-related signaling networks, including PI3K-Akt and NF-κB, while the epithelial-cell experiments provided supportive evidence for reduced inflammatory output and modulation of selected target-related genes. Overall, these findings provide an integrative framework for target prioritization and mechanistic exploration in COPD, rather than definitive mechanistic proof. Further validation in immune-cell and in vivo models will be required to confirm the predicted macrophage-centered mechanisms and their translational relevance.

## Figures and Tables

**Table 1:** Primer sequences used for RT-qPCR.

Gene	Primer Fwd (5′-3′)	Primer Rev (3′-5′)
ACTB	ACAGAGCCTCGCCTTTGC	ATCATCCATGGTGAGCTGGC
IKBKB	GATTGCCATCAAGCAGTGCC	TAAGCGCAGAGGCAATGTCA
PIK3CA	GGACCCGATGCGGTTAGAG	ATCAAGTGGATGCCCCACAG
IL6	CCACCGGGAACGAAAGAGAA	TCTCCTGGGGGTATTGTGGA
IL1B	CAGAAGTACCTGAGCTCGCC	AGATTCGTAGCTGGATGCCG
IL10	AAAGAAGGCATGCACAGCTC	TCGAAGCATGTTAGGCAGGT

## Data Availability

The data presented in this study are available within the article.

## Abbreviations

The following abbreviations are used in this manuscript:

ABCC1	ATP Binding Cassette Subfamily C Member 1
Akt	Protein Kinase B
ANOVA	Analysis of Variance
AUC	Area Under the Curve
BEAS-2B	Human Bronchial Epithelial Cell Line (BEAS-2B)
BP	Biological Process
CCK-8	Cell Counting Kit-8
CD163	CD163 Molecule
CIBERSORT	Cell-type Identification By Estimating Relative Subsets Of RNA Transcripts
COPD	Chronic Obstructive Pulmonary Disease
CTD	Comparative Toxicogenomics Database
DMEM	Dulbecco’s Modified Eagle Medium
DTS	Decision Tree (Classifier)
ELISA	Enzyme-Linked Immunosorbent Assay
GBM	Gradient Boosting Machine
GEO	Gene Expression Omnibus
GO	Gene Ontology
GlmBoost	Boosted Generalized Linear Model
GRN	Gene Regulatory Network
GSEA	Gene Set Enrichment Analysis
GROMACS	GROningen MAchine for Chemical Simulations
HPLC	High-Performance Liquid Chromatography
IKBKB	Inhibitor of Nuclear Factor Kappa B Kinase Subunit Beta (IKKβ)
IL-6	Interleukin-6
IL-8	Interleukin-8
IVW	Inverse-Variance Weighted
kb	Kilobase(s)
KEGG	Kyoto Encyclopedia of Genes and Genomes
KNN	k-Nearest Neighbor(s)
KO	Knockout
LASSO	Least Absolute Shrinkage and Selection Operator
LPS	Lipopolysaccharide
MAPK	Mitogen-Activated Protein Kinase
MD	Molecular Dynamics
MR	Mendelian Randomization
NF-κB	Nuclear Factor Kappa B
OMIM	Online Mendelian Inheritance in Man
PDB	Protein Data Bank
PI3K	Phosphoinositide 3-Kinase
PIK3CA	Phosphatidylinositol-4,5-Bisphosphate 3-Kinase Catalytic Subunit Alpha
PLIP	Protein–Ligand Interaction Profiler
RLS	Ridge Least Squares
RF	Random Forest
RMSD	Root-Mean-Square Deviation
RMSF	Root-Mean-Square Fluctuation
ROC	Receiver Operating Characteristic
r^2^	Squared Correlation Coefficient (LD r^2^)
SASA	Solvent-Accessible Surface Area
scRNA-seq	Single-Cell RNA Sequencing
SEA	Similarity Ensemble Approach
SHAP	Shapley Additive exPlanations
SNP	Single-Nucleotide Polymorphism
SPR	Surface Plasmon Resonance
SVM	Support Vector Machine
SVM-RFE	Support Vector Machine–Recursive Feature Elimination
TNF	Tumor Necrosis Factor
TNF-α	Tumor Necrosis Factor Alpha
UMAP	Uniform Manifold Approximation and Projection
XGBoost	eXtreme Gradient Boosting
